# Novel polyoxins generated by heterologously expressing polyoxin biosynthetic gene cluster in the *sanN* inactivated mutant of *Streptomyces ansochromogenes*

**DOI:** 10.1186/1475-2859-11-135

**Published:** 2012-10-08

**Authors:** Jine Li, Lei Li, Chi Feng, Yihua Chen, Huarong Tan

**Affiliations:** 1 State Key Laboratory of Microbial Resources, Institute of Microbiology, Chinese Academy of Sciences, Beijing, 100101, China; 2 Graduate School of Chinese Academy of Sciences, Beijing, 100039, China

**Keywords:** Polyoxin, Gene cluster, Heterologous expression, *S. ansochromogenes*

## Abstract

**Background:**

Polyoxins are potent inhibitors of chitin synthetases in fungi and insects. The gene cluster responsible for biosynthesis of polyoxins has been cloned and sequenced from *Streptomyces cacaoi* and tens of polyoxin analogs have been identified already.

**Results:**

The polyoxin biosynthetic gene cluster from *Streptomyces cacaoi* was heterologously expressed in the *sanN* inactivated mutant of *Streptomyces ansochromogenes* as a nikkomycin producer. Besides hybrid antibiotics (polynik A and polyoxin N) and some known polyoxins, two novel polyoxin analogs were accumulated. One of them is polyoxin P that has 5-aminohexuronic acid with *N*-glycosidically bound thymine as the nucleoside moiety and dehydroxyl-carbamoylpolyoxic acid as the peptidyl moiety. The other analog is polyoxin O that contains 5-aminohexuronic acid bound thymine as the nucleoside moiety, but recruits polyoximic acid as the sole peptidyl moiety. Bioassay against phytopathogenic fungi showed that polyoxin P displayed comparatively strong inhibitory activity, whereas the inhibitory activity of polyoxin O was weak under the same testing conditions.

**Conclusion:**

Two novel polyoxin analogs (polyoxin P and O) were generated by the heterologous expression of polyoxin biosynthetic gene cluster in the *sanN* inactivated mutant of *Streptomyces ansochromogenes*. Polyoxin P showed potent antifungal activity,while the activity of polyoxin O was weak. The strategy presented here may be available for other antibiotics producers.

## Introduction

**Figure 1:**
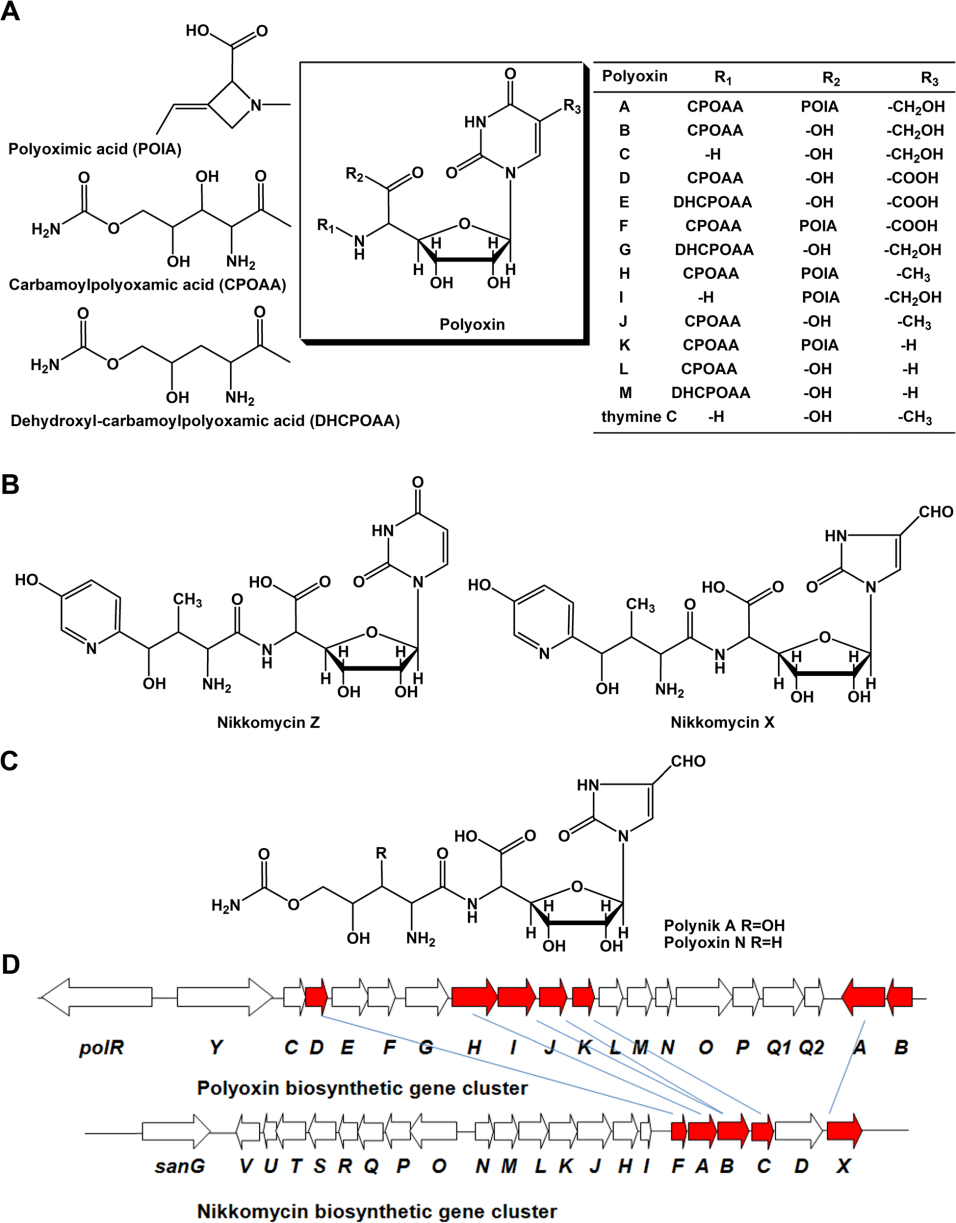
**Structures of polyoxins, nikkomycins, hybrid antibiotics and their biosynthetic gene clusters.A**, structures of polyoxin analogs; **B**, structures of two representative nikkomycins; **C**, structures of two hybrid antibiotics; **D**, gene organization of polyoxin and nikkomycin biosynthetic gene clusters. The red arrows indicate genes related to the nucleoside moiety biosynthesis, and the homolog genes are linked by oblique lines.

Polyoxins (A-M), a group of peptidyl nucleoside antibiotics (Figure 1), were isolated from the culture broth of *Streptomyces cacaoi*[1]. Due to their structural similarity to UDP-*N*-acetylglucosamine, polyoxins act as competitive inhibitors of chitin synthetases and display potent inhibitory activity against phytopathogenic fungi and insects [2-4]. Polyoxins were composed of nucleoside moiety and peptidyl moiety connected via a peptide bond. In different polyoxin analogs, the nucleoside moiety could be a 5-aminohexuronic acid with *N*-glycosidically bounded different bases, such as uracil, thymine, 5-carboxyl-uracil or 5-hydroxylmethyl-uracil; the peptidyl moiety includes C-5 linked carbamoylpolyoxamic acid (CPOAA) or dehydroxyl-carbamoylpolyoxamic acid (DHCPOAA) and C-6 linked polyoximic acid (POIA) [5-8]. Studies on the structure-activity relationship (SAR) of polyoxins showed that the thymine derived polyoxins (polyoxin H and J) have higher inhibitory activity of chitin synthetase compared with polyoxin A, B, D, F and L, indicating that thymine is better than the other bases at bioactivity [3].

The genes responsible for the biosynthesis of polyoxins have been cloned from *S. cacaoi*[9]. It was suggested that seven genes *polA, B, D, H, I, J* and *K* were involved in polyoxin nucleoside moiety biosynthesis (Figure 1). Among them, *polB*, a proposed thymidylate synthase coding gene, likely governs the methylation of C-5′ position of uracil to afford thymine base. Genes encoding enzymes for subsequent oxidation at C-7′ are out of the cloned polyoxin biosynthetic cluster and may locate at other positions of chromosome. Previous efforts of heterologously expressing the polyoxin biosynthetic gene cluster in *S. lividans* TK24 obtained only polyoxin H [9].

To expand the diversity of polyoxins by combinatorial biosynthetic strategy, the nikkomycin producer was considered as a host for heterologous expression of polyoxin. Nikkomycins (Figure 1) are peptidyl nucleoside antibiotics produced by *S. ansochromogenes* or *S. tendae*[10,11]. The peptidyl moiety of nikkomycin is hydroxypyridylhomothreonine; while its nucleoside moiety is 5-aminohexuronic acid *N*-glycosidically bounded uracil in nikkomycin Z (the same as polyoxin K, L and M) or 5-aminohexuronic acid *N*-glycosidically bounded imidazolone in nikkomycin X. The gene cluster responsible for biosynthesis of nikkomycins has already been cloned and sequenced from *S. ansochromogenes* (Figure 1) [12]. Previous combinatorial biosynthesis attempts resulted in polyoxin N and a novel compound polynik A by expressing the polyoxin peptidyl moiety genes in *sanN* inactivated mutant (ΔsanN) [13]. SanN catalyzes the conversion from benzoate-CoA to benzaldehyde, which is a precursor of nikkomycin peptidyl moiety [14]. The resulting ΔsanN strain lost its ability to synthesize hydroxypyridylhomothreonine, but it can accumulate 5-aminohexuronic acid bounded uracil or 5-aminohexuronic acid bounded imidazole. Considering the yield of nikkomycins produced by *S. ansochromogenes* is quite high [12], the ΔsanN strain can supply not only the 5-aminohexuronic acid bounded imidazole for hybrid polynik compounds but also 5-aminohexuronic acid bounded uracil for more polyoxin production, which may lead to the production of novel polyoxin analogs.

Therefore, we introduced the polyoxin biosynthetic gene cluster into the ΔsanN strain of *S. ansochromogenes* in this study. Here, we report that two new polyoxins (polyoxin O and P) were isolated from this constructed recombinant strain and the production of hybrid compounds, polynik A and polyoxin N, were also observed. Bioactive investigations revealed that polyoxin P displayed strong antifungal activity, whereas polyoxin O displayed weak antifungal activity. The strategy we have taken in this study is significant to obtain novel antibiotics or their derivatives with potential value in application.

## Results

### Construction of *S. ansochromogenes* ΔsanN/pPOL

Cosmid 9A, containing the clustered polyoxin biosynthetic genes, was screened by PCR from the genomic cosmid library preserved in our lab. To construct a recombinant plasmid for heterologous expression of genes, the clustered polyoxin genes in cosmid 9A were transferred to pSET152 via Red/ET recombination technology to result in pPOL, which could be integrated into the chromosome in *Streptomyces*.

Plasmid pPOL was introduced into the ΔsanN strain of *S. ansochromogenes* by *E. coli-Streptomyces* intergenic conjugation, the resulting conjugates were verified by PCR (Additional file 1: Figure S1). The exconjugate ΔsanN/pPOL was then incubated in SP liquid medium. After 5 days, the fermentation broth was used to detect bioactivity against phytopathogenic fungus. Growth inhibition of *A. kikuchiana* was clearly observed (Additional file 1: Figure S2)*.*

### Isolation and characterization of polyoxin P

**Figure 2:**
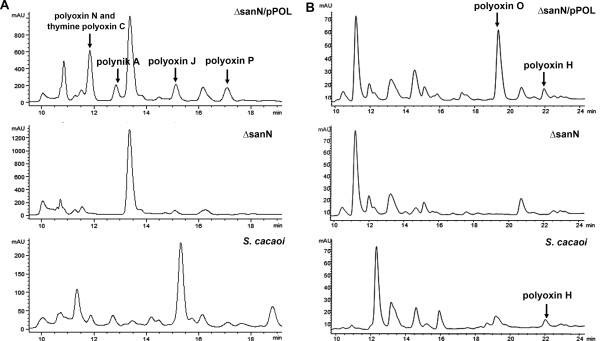
**HPLC analyses of the antibiotics produced by ΔsanN/pPOL.** The corresponding peaks of different compounds are marked by arrows. **A**, HPLC traces identifying as polyoxin P; **B**, HPLC traces identifying as polyoxin O. Polyoxin N and thymine polyoxin C had the similar retention time at about 11.8 min, the retention times of polynik A, polyoxin J and P were 12.9 min, 15.2 min and 17.2 min, respectively. The retention times for polyoxin O and H were about 19.5 min and 22 min, respectively.

**Figure 3:**
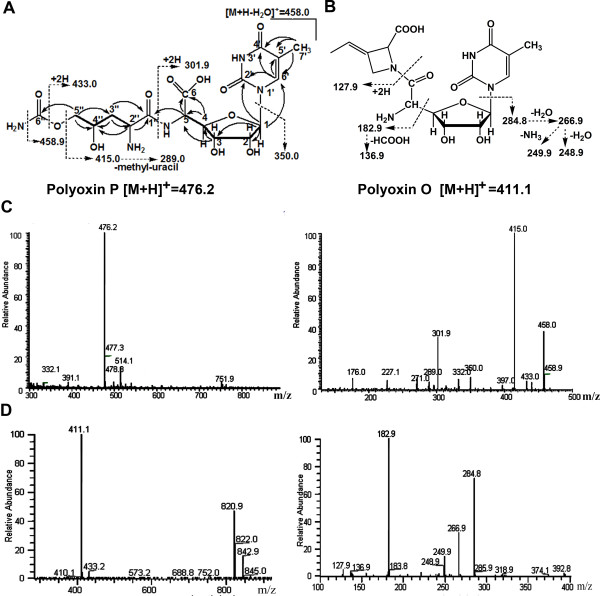
**MS and NMR analyses of polyoxin P and O.A**, structure of polyoxin P. The fragmentation pattern of MS/MS is marked in dash lines. The bold lines indicate COSY correlations, and the HMBC correlations are showed by arrows; **B**, structure of polyoxin O. The fragmentation pattern of MS/MS is marked in dash lines; **C**, MS and MS/MS spectra of polyoxin P; **D**, MS and MS/MS spectra of polyoxin O.

To identify the antifungal antibiotics produced by the recombinant strain ΔsanN/pPOL, the fermentation broth was processed by using a macroporous absorption resin HP-20 column. Then, it was subjected to HPLC analysis (Figure 2). Compared with the host strain, the newly emerged peaks in the recombinant strain were collected and further analyzed by LC-MS and MS/MS (Additional file 1: Figure S3). As anticipated, the hybrid compounds polynik A and polyoxin N were observed along with several known polyoxins including thymine polyoxin C and polyoxin J. Besides those, a new compound with retention time at 17.1 min was discovered. Preliminary MS analysis afforded an [M+H]^+^ ion at *m/z* 476.2 (Figure 3), which was inconsistent with any known polyoxins. The new compound was designated as polyoxin P. The production of polyoxin P is about 90 μg ml^-1^.

Polyoxin P was then purified by HPLC and further analyzed by high resolution electrospray ionization mass spectrometry (HR-ESI-MS) and NMR. HR-ESI-MS results showed an [M+H]^+^ ion at *m/z* 476.1647 (Additional file 1: Figure S4), corresponding to the molecular formula C_17_H_25_O_11_N_5_ (476.1629 calculated). Subsequent MS/MS fragmentation pattern indicated that the polyoxin P contains a thymine base and DHCPOAA as peptidyl moiety (Figure 3). Based on the NMR results (Table 1 and Additional file 1: Figure S5), the chemical structure of polyoxin P was determined (Figure 3). The C-7′ methyl group can be assigned by the correlation from H-7′ (1.828 ppm) to C-5′ (111.7 ppm); and the DHCPOAA moiety can be determined by ^1^H, ^13^C, COSY and HMBC data. Structural elucidation of polyoxin P adds a new member of polyoxins with thymine besides polyoxin H and J.

**Table 1:** ^**1**^**H and**^**13**^**C NMR data of polyoxin P**

**Position**	^**1**^**H (δ, mult.,*J*)**	^**13**^**C (δ)**
1	5.734(1H, d, 4.8)	90.6
2	4.366(1H, m)	72.2
3	4.467 (1H, m)	69.9
4	4.224(1H, m)	82.5
5	4.780(1H, d, 4.2)	54.4
6		171.4
2′		151.7
4′		166.4
5′		111.7
6′	7.331(1H, s)	138.2
7′	1.828 (3H, s)	11.5
1″		169.4
2″	4.194(1H, t, 7.2)	51.7
3″	1.919- 2.107(2H, m)	33.5
4″	4.097-4.086(1H, m)	66.8
5″	3.926-4.030(2H, m)	68.1
6″		159.1

*Polyoxin P in D_2_O,*δ* in ppm, *J* in Hz.

### Isolation and characterization of polyoxin O

The fermentation broth of ΔsanN/pPOL strain was processed and subjected to HPLC analysis. Two compounds with retention time at about 19.4 and 22 min were distinguished in comparison with the host strain as a control (Figure 2). The compounds were collected and subjected to LC-MS and MS/MS analysis (Figure 3). Results showed that the compound has [M+H]^+^ ion at m/z 411.1, and contains aminohexuronic acid with N-glycosidically bound thymine as the nucleoside moiety, polyoximic acid as the peptidyl moiety (Figure 3). So far as we know, this compound has not been reported, and was named as polyoxin O. In contrast to the standard compound, the peak at 22 min was identified as polyoxin H.

### Bioassay of polyoxin P and polyoxin O

**Figure 4:**
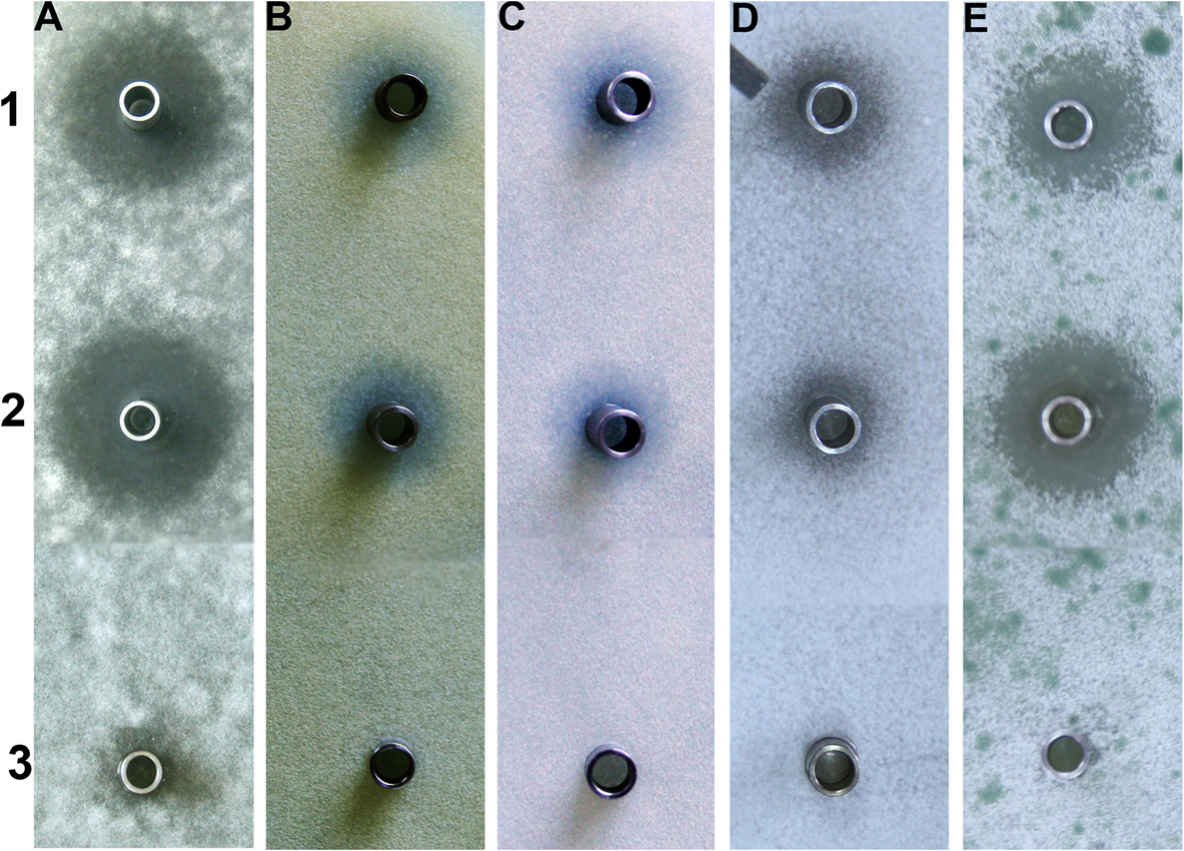
**Bioassay of polyoxin H, polyoxin P and polyoxin O against fungi.A**, *Alternaria kikuchiana*; **B**, *Aspergillus fumigates*; **C,***Rhizoctonia solani*; **D**, *Botrytis cinerea*; **E**, *Trichoderma viride.* 1, polyoxin H; 2, polyoxin P; 3, polyoxin O.

To characterize the antifungal activities of the two novel polyoxins, polyoxin P and O were subjected to bioassay against *Alternaria kikuchiana, Aspergillus fumigates, Rhizoctonia solani, Botrytis cinerea* and *Trichoderma viride* using polyoxin H as a control (Figure 4)*.* It was shown that polyoxin P displayed potent inhibitory activity, whereas the activity of polyoxin O was very weak.

## Discussion

Given more and more biosynthetic machineries of natural products are elucidated, combinatorial biosynthesis has been developed as an optional way to generate novel antibiotics. A large number of successful examples demonstrated that the method was available [15-17]. In this study, we constructed a ΔsanN/pPOL strain that provides a 5-aminohexuronic uracil and 5-aminohexuronic imidazolone as precursors for novel polyoxins formation. Two novel polyoxins (polyoxin P and O) were obtained together with two hybrid compounds (polynik A and polyoxin N) and several known polyoxins (polyoxin H, J and thymine polyoxin C).

All the uracil derived polyoxins accumulated in the ΔsanN/pPOL were added a C-5′ methyl to form thymine base, which should be catalyzed by the thymidylate synthase PolB. Since the genes encoding enzymes to modify C-7′ methyl group into hydroxylmethyl and carboxylic acid are not in pPOL, ΔsanN/pPOL is an ideal strain to produce thymine derived polyoxins. Previous SAR (structure-activity relationships) research revealed that thymine derived polyoxins have better antifungal activity [3]. The bioassay against phytopathogenic fungi showed that the polyoxin P possesses comparatively strong bioactivity as polyoxin H (Figure 4), while polyoxin O has very weak bioactivity, indicating that components containing different peptidyl moieties possess different biological activity (DHCPOAA is important, whereas POIA is not necessary).

Our results proved that the *S. ansochromogenes* has capacity to supply enough 5-aminohexuronic uracil for polyoxin biosynthesis. To generate more thymine derived polyoxins, blocking the 5-aminohexuronic imidazolone pathway and increasing the production of polyoxin peptidyl moieties are considered to optimize this heterologous expression system. The strategy in this study is efficient to obtain novel antibiotics or their derivatives and it may be available for other bacteria to result in new compounds.

## Conclusion

Including two new members (polyoxin P and O), variety of thymine derived polyoxins and hybrid compounds were generated by hererologous expression of polyoxin biosynthetic gene cluster in the *sanN* disruption mutant of *S. ansochromogenes*. Polyoxin P showed inhibitory activity against *A. kuchiana, A. fumigates, R. solani, B. cinerea* and *T. viride,* whereas the bioactivity of polyoxin O was quite weak against above five indicator strains under the same testing conditions. The strategy presented here may be available for other antibiotics producers.

## Materials and methods

### Stains and culture conditions

For spores collection, *S. cacaoi* subsp. *asoensis* AS4.1602 and *S. ansochromogenes sanN* disruption mutant were grown on mannitol/soya medium (MS) and minimal medium (MM), respectively [18]. For genomic DNA extraction, *Streptomyces* were grown in liquid YEME medium containing 20% sucrose, which was also used as seed culture for fermentation. For antibiotics production, the seed culture was inoculated into fresh SP medium at 1% ratio and further incubated for 5 days at 28°C, and then the culture broth was collected. *Alternaria kikuchiana, Aspergillus fumigates, Rhizoctonia solani, Botrytis cinerea* and *Trichoderma viride* obtained from the China General Microbiological Culture Collection Center (CGMCC) were grown at 28°C and used as indicator strains for bioassay. *E. coli* JM109 and DH5α were used as the hosts for propagating plasmids. Methylation-deficient *E. coli* ET12567 (pUZ8002) was used as a host for transferring DNA from *E. coli* to *Streptomyces* by intergeneric conjugation [18].

When necessary, antibiotics were used at the following final concentrations: 100 μg ml^-1^ ampicillin (Amp), 100 μg ml^-1^ kanamycin (Kan), 100 μg ml^-1^ apramycin (Apr), 15 μg ml^-1^ tetracycline and 12.5 μg ml^-1^ chloramphenicol in LB for *E. coli*; 10 μg ml^-1^ Apr or Kan in MM, MS and YEME for *Streptomyces*.

### Plasmids and DNA manipulations

Isolation of plasmids and chromosomal DNA were carried out according to the standard methods [18,19]. Conjugal transfer from *E. coli* to *Streptomyces* and Red/ET recombination were performed as described elsewhere [18,20]. Cosmid 9A containing the polyoxin biosynthetic gene cluster was screened by PCR from the genomic cosmid library preserved in our lab. Then, cosmid cos9A was digested with *Bam*HI and the resulting 10.6 kb lineared vector fragment was self-ligated to create cosAfl. CosAfl was digested with *Eco*RI and the excised DNA fragment (about 4.1 kb) was inserted into the same site of pSET152 to generate pSET152::Afl. Plasmid pSET152::*polR*[21,22] was digested with *Eco*RI/*Xba*I and the 3.4 kb DNA fragment containing *polR* was inserted into pIJ2925 to give pIJ2925::*polR*. A 3.4 kb *Xba*I/*Bgl*II DNA fragment was excised from pIJ2925::*polR* and then inserted into the *Xba*I/*Bam*HI site of pSET152::Afl to result in pSET152::*polR*::Afl. pSET152::*polR*::Afl was linearized with *Nde*I/*Spe*I digestion and introduced into *E. coli* BW25113/pIJ790 containing cosmid cos9A. Then pPOL was generated after λ-Red-mediated recombination.

### Detection of polyoxins and antifungal bioassays

The fermentation broth was chromatographed on a macroporous absorption resin HP-20 column, and the flow-through was collected and concentrated in vacuum. Then, 6 volumes of cold ethanol were added and kept at 4°C overnight, the precipitate was collected by centrifugation and dried at room temperature. The crude powder was dissolved in water. Polyoxins are monitored by HPLC analysis with Agilent 1100 HPLC system. Two different HPLC conditions were used to identify different compounds. For detection of polyoxin P: ZORBAX SB-AQ was used as the column, 95% H_2_O (containing 0.1% trifluoroacetic acid) and 5% methanol were used as the mobile phase, the flow rate was 0.3 ml/min, and the absorption wavelength was 260nm. For detection of polyoxin O: ZORBAX SB-C18 column was used for analysis, and mobile phase conditions were the same as those for detecting nikkomycins described elsewhere [23]. The detection wavelength was 260 nm. Bioassay against *A. kikuchiana*, *A. fumigates, R. solani, B. cinerea* and *T. viride* was carried out according to the method used in previous study [24], and 100μl polyoxins solution (about 100 μg ml^-1^) was added.

### Isolation of polyoxins

The method for isolating polyoxin P was similar to that for polynik A [13]. The fermentation broth was harvested by centrifugation and the pH was adjusted to 4.5 with acetic acid. Then, it was chromatographed on a macroporous absorption resin HP-20 (Mitsubishi) column, and the flow-through was collected and subjected to a Dowex 50WX2 (Sigma) column. The column was eluted with 0.4 N ammonia solution and the samples with antifungal activity was collected and concentrated to a small volume (less than 10 ml). About 6 volumes of cold ethanol were added and the precipitated sample was dried and dissolved in water, subsequently it was further purified by HPLC and identified as a pure polyoxin P. Polyoxin O was first purified by the ion pair HPLC program [23], lyophilized and dissolved in water. The sample was subsequently subjected to HPLC to remove salts with following conditions: ZORBAX SB-C18 was used as the column with 1 ml min^-1^ flow rate at 40°C, a liner gradient of 10%-50% solution B (solution A = 0.1% trifluoroacetic acid; solution B = methanol) over 30 min was used as the elution profile.

### Spectrometric analyses

MS and Tandem Mass Spectrometry analyses were carried out on LCQ Deca XP Plus (Thermo-Finnigan) with the electrospray ionization source in positive mode. For high resolution mass spectrometry analysis, an Aligent 1200 HPLC system and 6520 QTF-MS system were used in positive mode. NMR analyses were subjected with a Bruker spectrometer (AV600 MHz).

## Competing interests

The authors declare that they have no competing interests.

## Authors’ contributions

JL and LL carried out experiments, analyzed the primary data and wrote the draft manuscript. CF assisted with experiments. YC assisted with data analysis and revised the manuscript. HT supervised the whole research work and revised the manuscript. All authors read and approved the final manuscript.

## Supplementary Material

Additional file 1 **Figure S1. Analysis of conjugated ΔsanN/pPol strains by PCR.** A, RTS-afsR (5’-CCTCTACCGCAGTCTCCT-3’) and RTA-afsR (5′-TGTCCTCGTCCTCCAGTT-3′) were used as primers for PCR amplification; B, RTS-26 (5′-CCGCTCGCTCCACATCAAC-3′) and RTA-26 (5′-AGCCAGGAGTGGGTGAGGT-3′) were used as primers for PCR amplification. Lane 1, *S. cacaoi*;lane 2, *S. ansochromogenes* 7100; lanes 3-8, different clones from conjugated ΔsanN/pPol;lane 9, ΔsanN mutant; lane 10, DNA marker. Figure S2, Bioassay of the fermentation broth of Δ*sanN*/pPOL. 1, the fermentation broth of *S. ansochromgenes* 7100; 2, the fermentation broth of *sanN* disruption mutant; 3, the fermentation broth of *S. cacaoi*; 4-9, the fermentation broth of Δ*sanN*/pPOL. Figure S3, MS and MS/MS spectra of polyoxin N, polynik A, polyoxin J and thymine-polyoxin C. A, MS and MS/MS spectra of polyoxin N; B, MS and MS/MS spectra of polynik A; C, MS and MS/MS spectra of polyoxin J; D, MS and MS/MS spectra of thymine polyoxin C. Figure S4, HR-ESI-MS spectrum of polyoxin P. Figure S5, NMR spectrum of polyoxin P. A, The 1H-NMR spectrum of polyoxin P; B, The 13C-NMR spectrum of polyoxin P; C, The DEPT spectrum of polyoxin P; D, The COSY spectrum of polyoxin P; E, The HMQC spectrum of polyoxin P; F, The HMBC spectrum of polyoxin P. Click here for file
