# Supplementary material for: Novel polyoxins generated by heterologously expressing polyoxin biosynthetic gene cluster in the sanN inactivated mutant of Streptomyces ansochromogenes
Source: Microb Cell Fact. 2012 Oct 8;11:135. doi: 10.1186/1475-2859-11-135 (PMC3520715; doi:10.1186/1475-2859-11-135)

Supplementary data

**Novel polyoxins generated by heterologously expressing polyoxin biosynthetic gene cluster in the *∆sanN* mutant of *Streptomyces ansochromogenes***

Jine Li^1,#^, Lei Li^1,#^, Chi Feng^1,2^, Yihua Chen^1^ and Huarong Tan^1*^

**Supporting figures legends**

Sfig.1, Analysis of conjugated ∆sanN/pPol strains by PCR. A, RTS-afsR (5’-CCTCTACCGCAGTCTCCT-3’) and RTA-afsR (5’-TGTCCTCGTCCTCCAGTT-3’) were used as primers for PCR amplification; B, RTS-26 (5’-CCGCTCGCTCCACATCAAC-3’) and RTA-26 (5’-AGCCAGGAGTGGGTGAGGT-3’) were used as primers for PCR amplification. Lane 1, *S. cacaoi*；lane 2, *S. ansochromogenes* 7100； lanes 3-8, different clones from conjugated ∆sanN/pPol；lane 9, ∆sanN mutant; lane 10, DNA marker.

Sfig. 2, Bioassay of the fermentation broth of ∆*sanN*/pPOL. 1, the fermentation broth of *S. ansochromgenes* 7100; 2, the fermentation broth of *sanN* disruption mutant; 3, the fermentation broth of *S. cacaoi*; 4-9, the fermentation broth of ∆*sanN*/pPOL.

Sfig. 3, MS and MS/MS spectra of polyoxin N, polynik A, polyoxin J and thymine-polyoxin C. A, MS and MS/MS spectra of polyoxin N; B, MS and MS/MS spectra of polynik A; C, MS and MS/MS spectra of polyoxin J; D, MS and MS/MS spectra of thymine polyoxin C.

Sfig . 4, HR-ESI-MS spectrum of polyoxin P.

Sfig. 5, NMR spectrum of polyoxin P. A, The 1H-NMR spectrum of polyoxin P; B, The 13C-NMR spectrum of polyoxin P; C, The DEPT spectrum of polyoxin P; D, The COSY spectrum of polyoxin P; E, The HMQC spectrum of polyoxin P; F, The HMBC spectrum of polyoxin P.

Sfig. 1


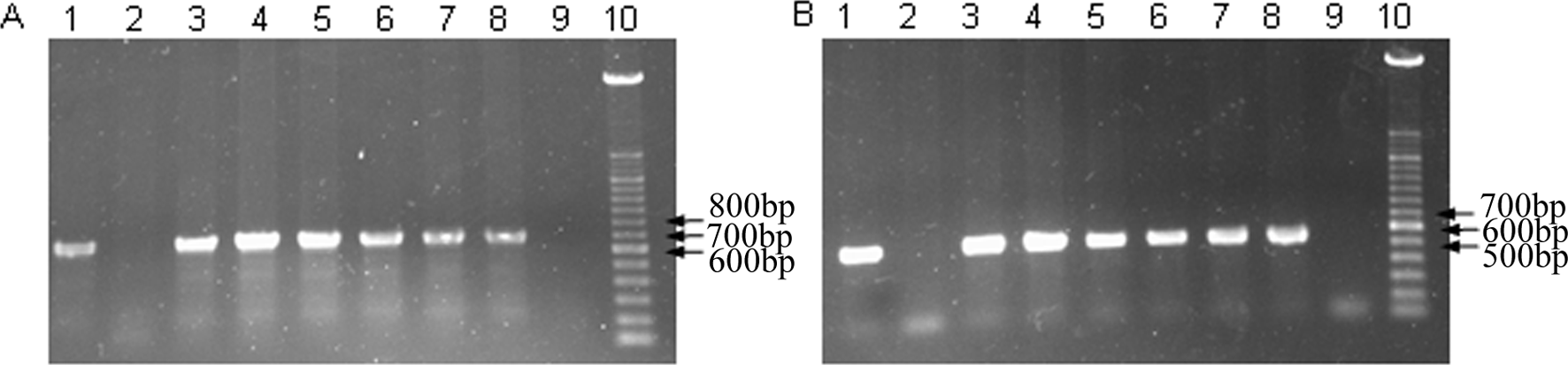


Sfig. 2


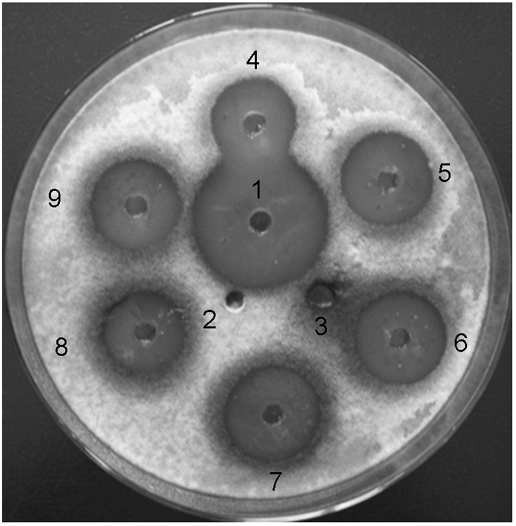


Sfig. 3

A
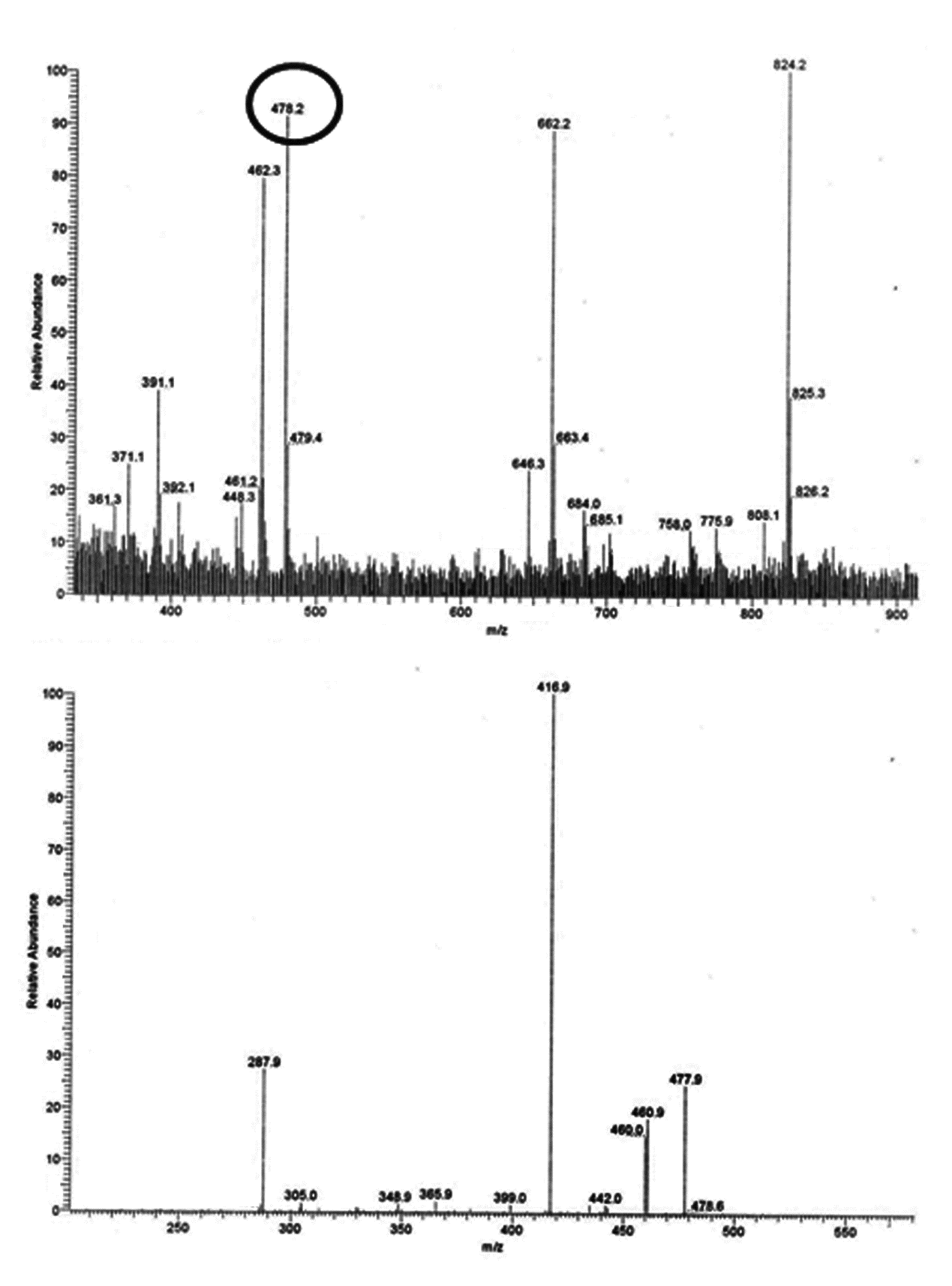


B


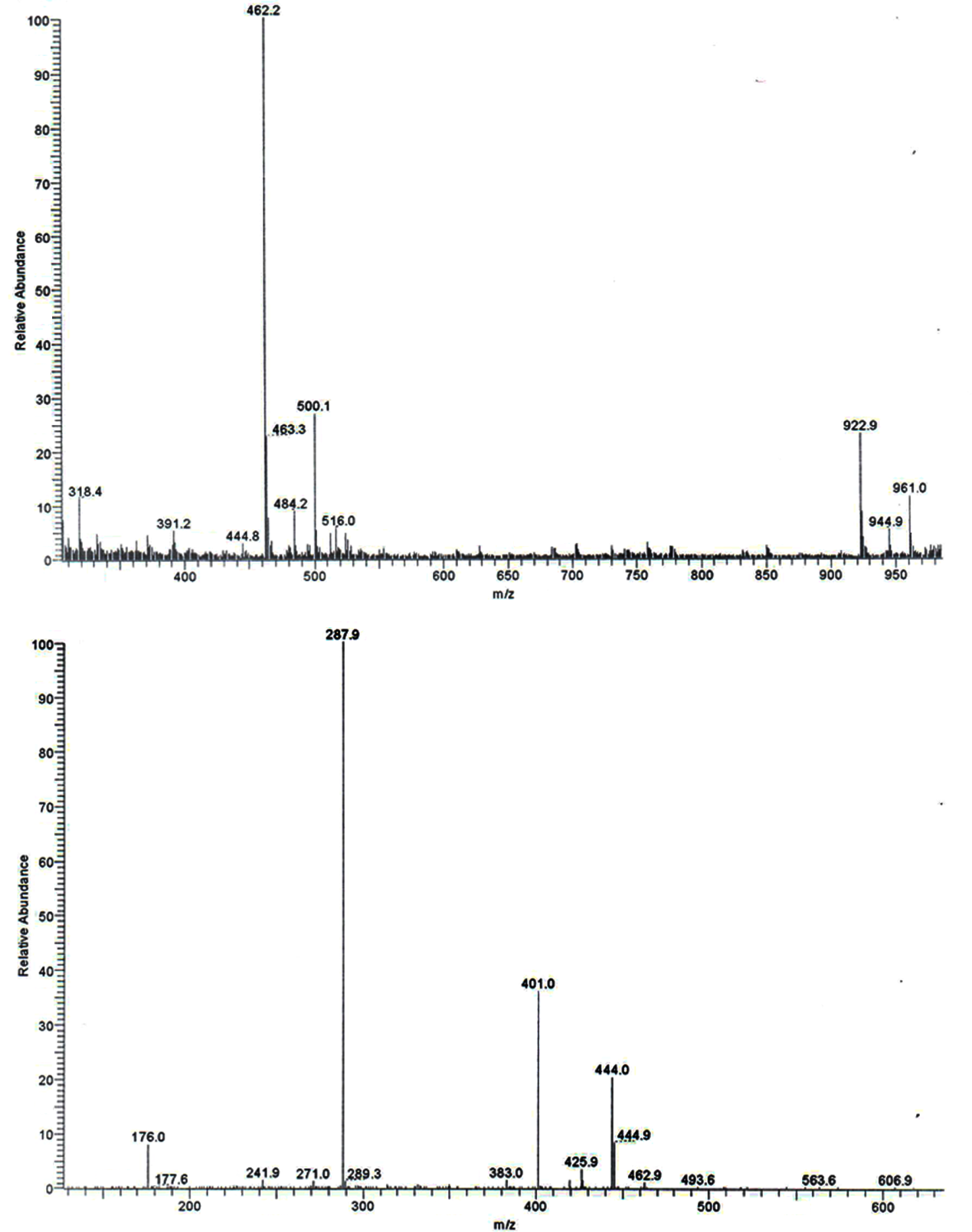


C
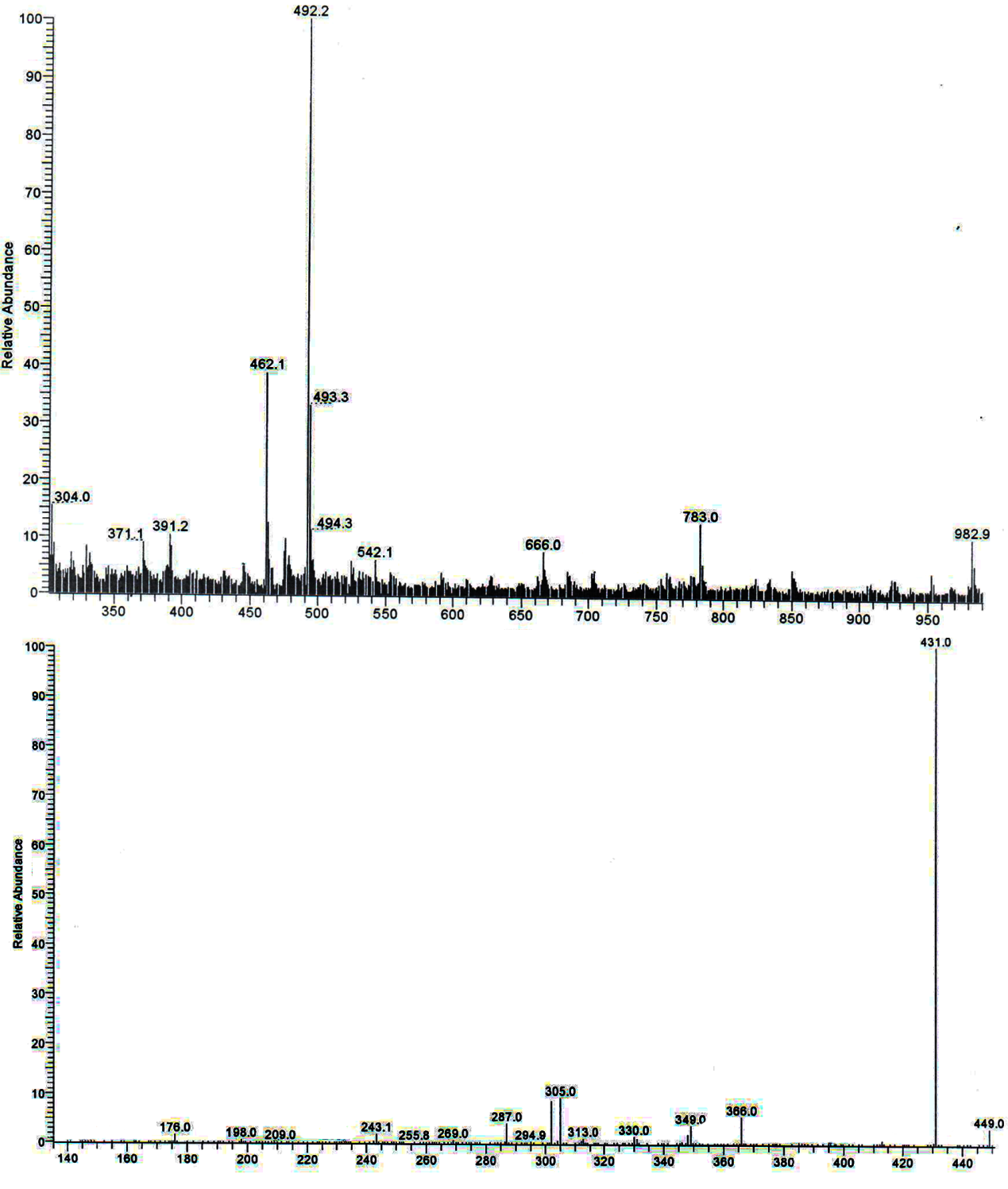


D
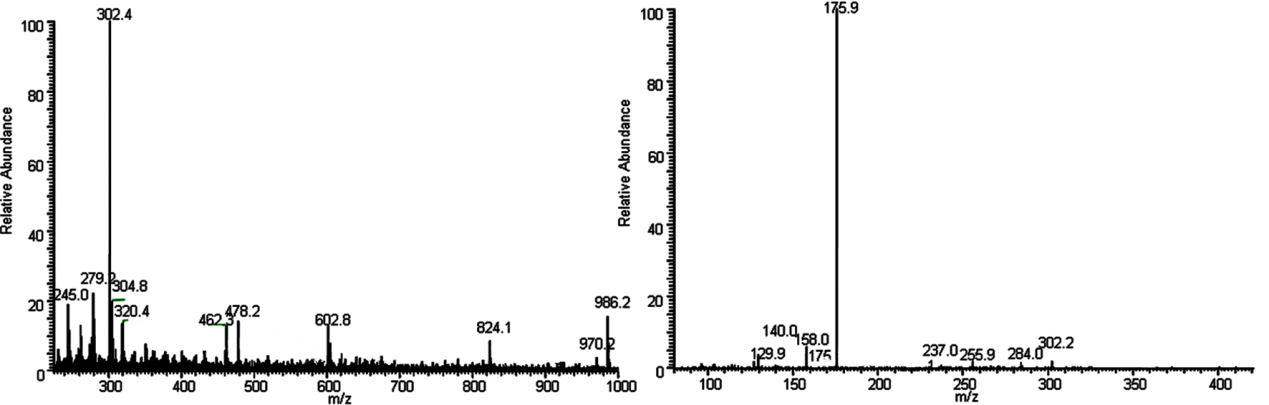


Sfig. 4


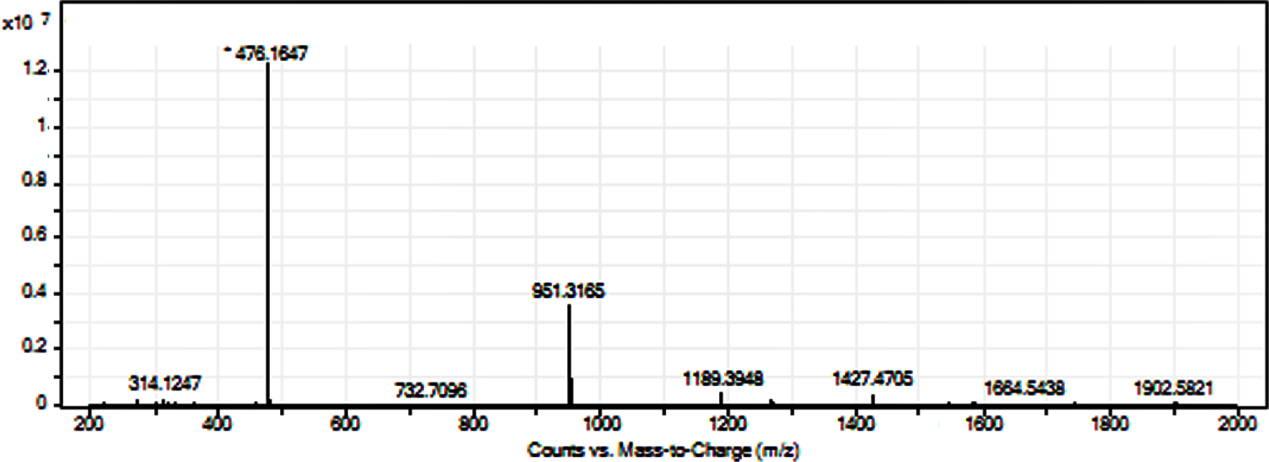


Sfig. 5

A


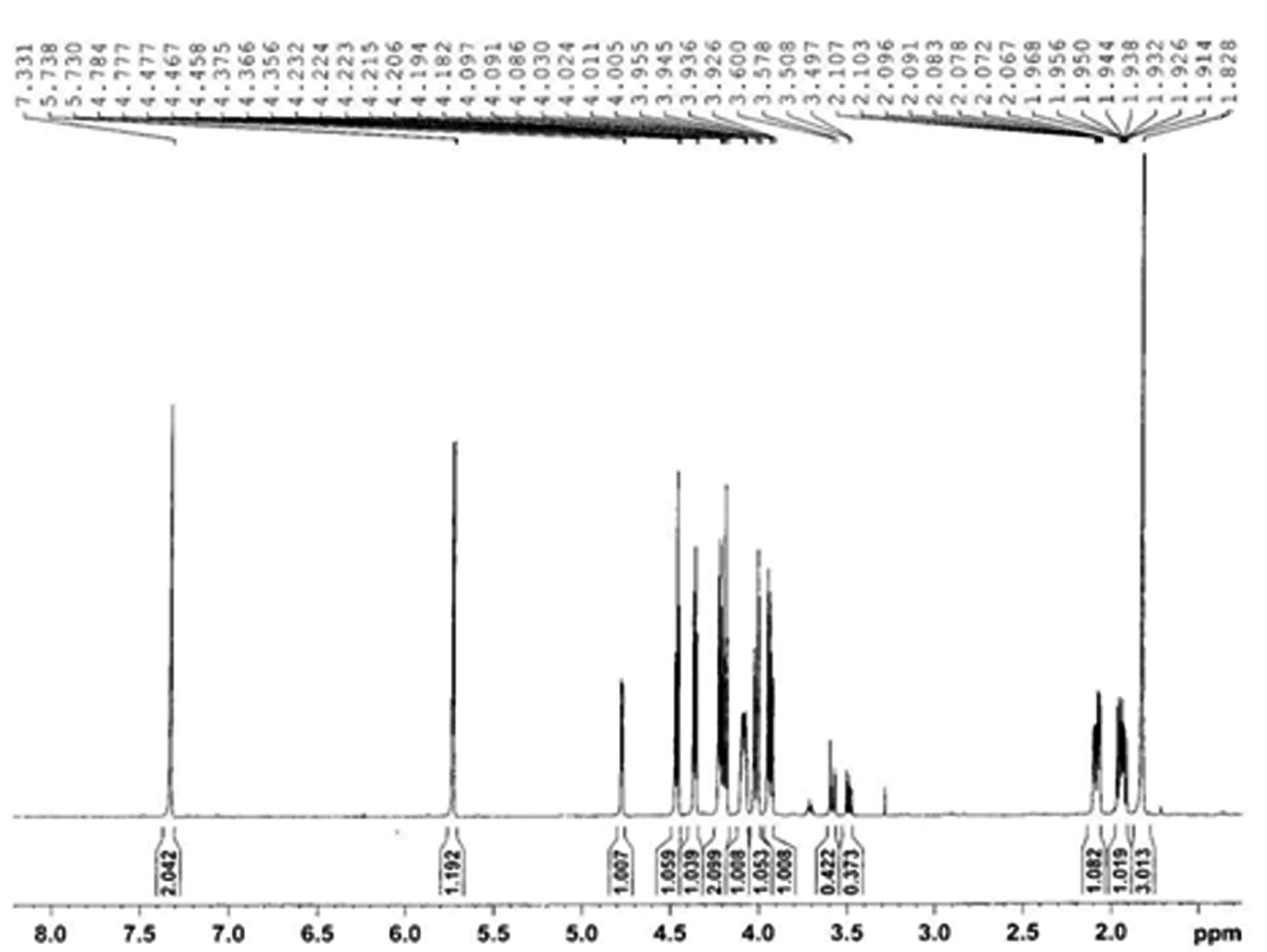


B


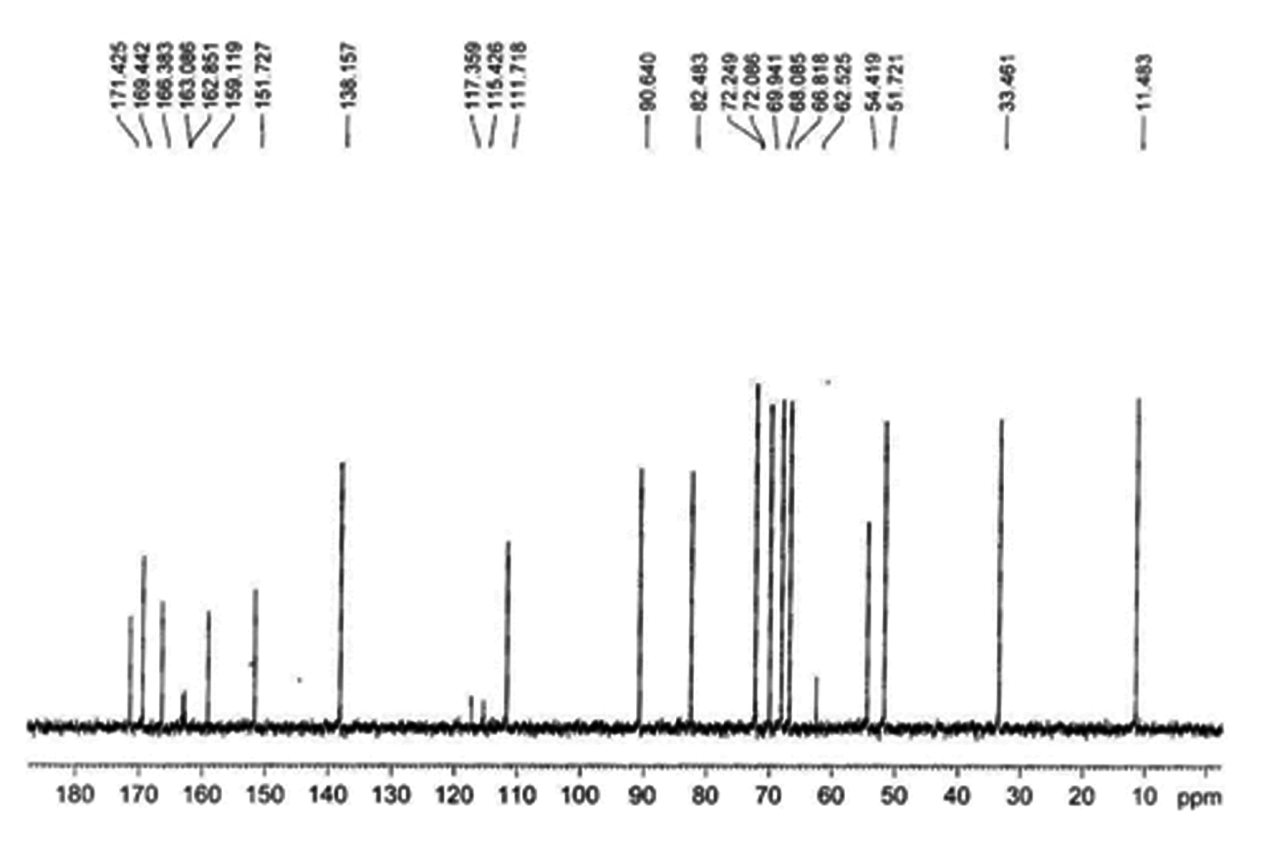


C


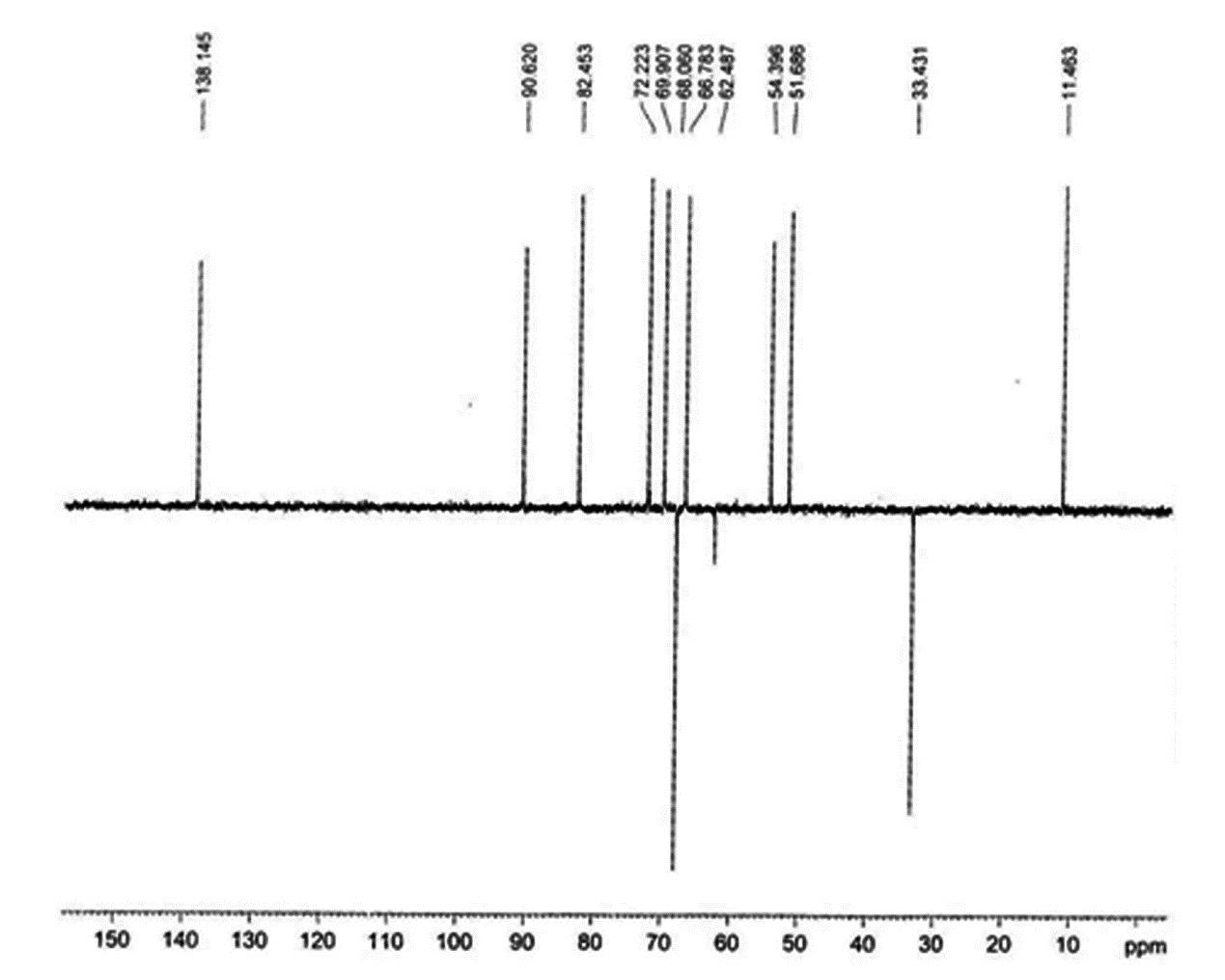


D


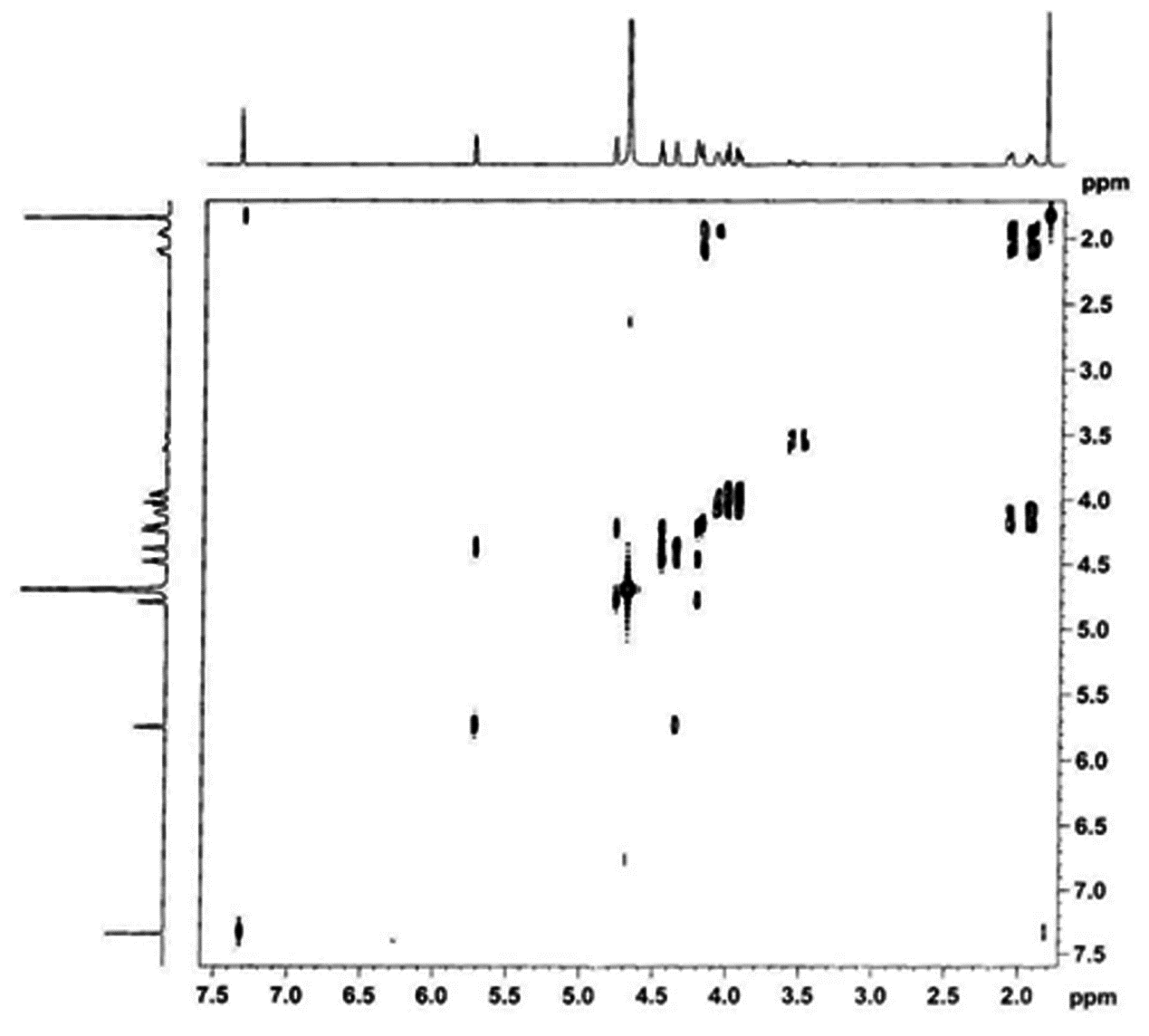


E


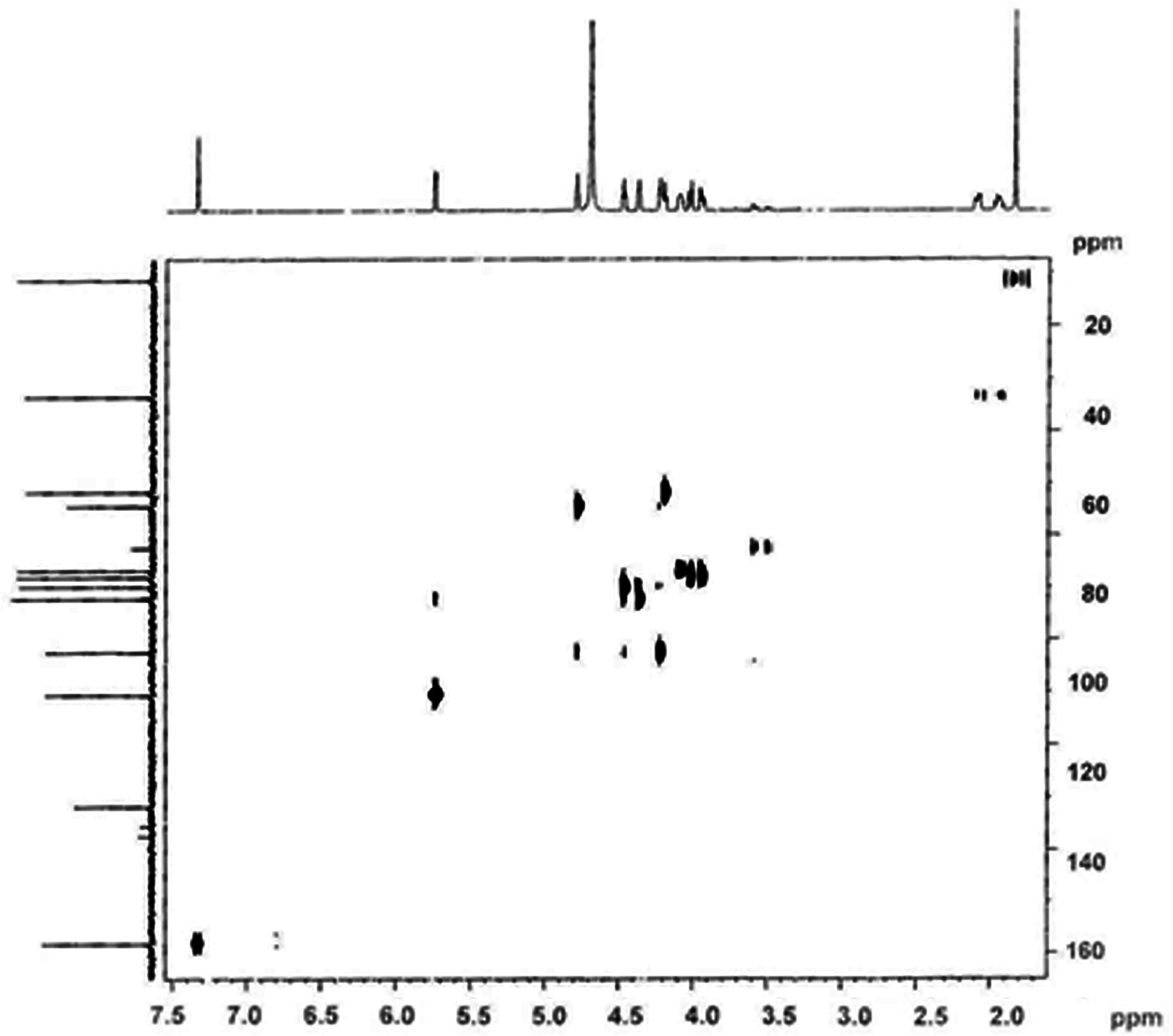


F


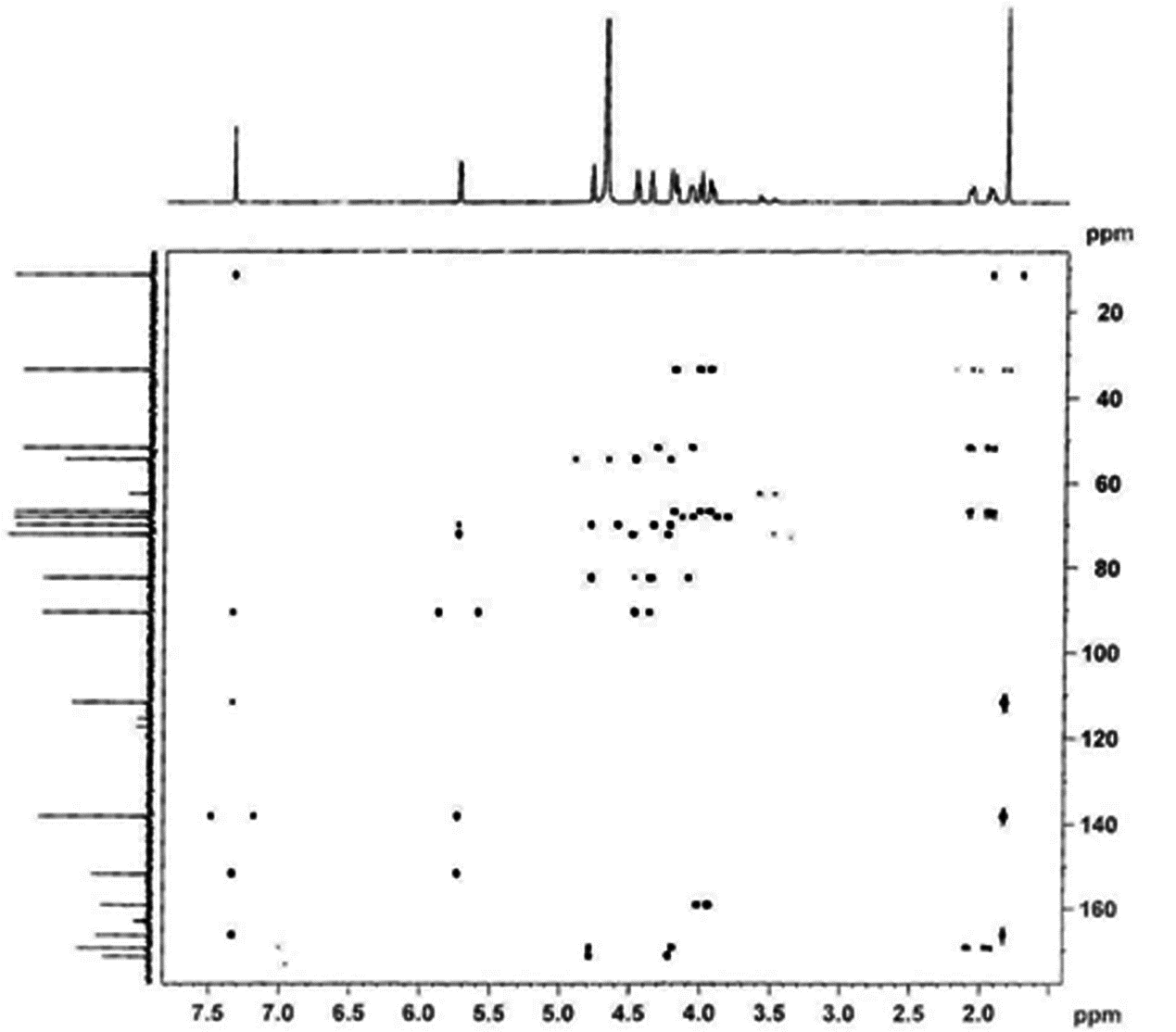

Supplement: Additional file 1 — Figure S1. Analysis of conjugated ΔsanN/pPol strains by PCR. A, RTS-afsR (5’-CCTCTACCGCAGTCTCCT-3’) and RTA-afsR (5′-TGTCCTCGTCCTCCAGTT-3′) were used as primers for PCR amplification; B, RTS-26 (5′-CCGCTCGCTCCACATCAAC-3′) and RTA-26 (5′-AGCCAGGAGTGGGTGAGGT-3′) were used as primers for PCR amplification. Lane 1, S. cacaoi;lane 2, S. ansochromogenes 7100; lanes 3-8, different clones from conjugated ΔsanN/pPol;lane 9, ΔsanN mutant; lane 10, DNA marker. Figure S2, Bioassay of the fermentation broth of ΔsanN/pPOL. 1, the fermentation broth of S. ansochromgenes 7100; 2, the fermentation broth of sanN disruption mutant; 3, the fermentation broth of S. cacaoi; 4-9, the fermentation broth of ΔsanN/pPOL. Figure S3, MS and MS/MS spectra of polyoxin N, polynik A, polyoxin J and thymine-polyoxin C. A, MS and MS/MS spectra of polyoxin N; B, MS and MS/MS spectra of polynik A; C, MS and MS/MS spectra of polyoxin J; D, MS and MS/MS spectra of thymine polyoxin C. Figure S4, HR-ESI-MS spectrum of polyoxin P. Figure S5, NMR spectrum of polyoxin P. A, The 1H-NMR spectrum of polyoxin P; B, The 13C-NMR spectrum of polyoxin P; C, The DEPT spectrum of polyoxin P; D, The COSY spectrum of polyoxin P; E, The HMQC spectrum of polyoxin P; F, The HMBC spectrum of polyoxin P. [file 1475-2859-11-135-S1.docx]
